# Development and validation of the caregiver-report version of the international grief questionnaire (IGQ-CG): Results from a Ukrainian sample of parents

**DOI:** 10.1177/13591045241260897

**Published:** 2024-06-13

**Authors:** Enya Redican, Mark Shevlin, Philip Hyland, Thanos Karatzias, Dmytro Martsenkovskyi, Menachem Ben-Ezra

**Affiliations:** 1 School of Psychology, 2596 Ulster University, Northern Ireland; 2 Department of Psychology, 8798 Maynooth University, Ireland; 3 School of Health & Social Care, 3121 Edinburgh Napier University, UK; 4 Department of Psychiatry and Narcology, Bogomolets National Medical University, Ukraine; 5 Institute of Psychiatry, Forensic Psychiatric Examination and Drug Monitoring of Ministry of Health of Ukraine, Ukraine; 6 School of Social Work, 42732 Ariel University, Israel

**Keywords:** prolonged grief disorder, ICD-11, validity, reliability, children and adolescents

## Abstract

The International Grief Questionnaire (IGQ) is a self-report measure of ICD-11 Prolonged Grief Disorder (PGD) in adults. This study sought to develop and validate a caregiver-report version of the IGQ for children and adolescents aged 7–17 years; the IGQ-Caregiver Version (IGQ-CG). 639 parents living in Ukraine provided data on themselves and one child in their household as part of the “*The Mental Health of Parents and Children in Ukraine Study: 2023 Follow-up”* study. The latent structure of the scale was tested using confirmatory factor analysis (CFA), while convergent validity was assessed through associations with other mental health correlates. Prevalence rates of probable ICD-11 PGD were estimated. CFA results supported a correlated two-factor model (‘core’ and ‘associated’ symptoms) and the internal reliability of the scale scores were acceptable. Convergent validity was supported through significant correlations with internalizing symptoms, while contact with the deceased, time since bereavement, and parental PGD were associated with higher scores on the IGQ-CG latent variables. The prevalence of probable ICD-11 PGD was 1.4%, and amongst those with a lifetime bereavement, the conditional rate was 3.2%. The IGQ-CG produces reliable and valid scores for ICD-11 PGD symptoms in children and adolescents as reported by their caregivers.

## Plain language summary

Prolonged Grief Disorder (PGD) is recognized as a grief-specific disorder included in the ICD-11. The International Grief Questionnaire (IGQ) is available to assess ICD-11 PGD symptoms in adults. However, there is no self-report measure available to assess ICD-11 PGD symptoms in children and adolescents. The researchers developed and validated a caregiver-report version of the IGQ for children and adolescents; the IGQ-Caregiver Version (IGQ-CG). Researchers found that the IGQ-CG was an accurate and consistent measure of PGD. Results showed that 1.4% of the sample met diagnostic requirements for ICD-11 PGD. The newly developed IGQ-CG can assist in the assessment and treatment of ICD-11 PGD in children and young people.

**Key points and relevance**

• Since Russia’s invasion of their country, children in Ukraine have endured a great deal of grief and loss.
• The International Grief Questionnaire (IGQ) has been developed as a self-report measure of ICD-11 Prolonged Grief Disorder.
• This study sought to develop and validate a caregiver-report version of the IGQ, named as the IGQ-Caregiver Version (IGQ-CG).
• Findings from this study provide support for the validity and reliability of the IGQ-CG.

The death of a loved one is a near universal experience that can affect anyone at any age. Bereavement is common among young people with almost half of all young people having experienced the death of a parent, sibling, grandparent, or other close family member by the age of eight (Paul & Vaswani, 2020). Moreover, in times of crisis and conflict young people often lose a loved one under particularly traumatic circumstances. For instance, since Russia’s invasion of their country, children in Ukraine have endured a great deal of grief and loss (UNICEF, 2023). An estimated 9,701 people have died since the conflict began, 555 of them being children (United Nations, 2023), and when military casualties are taken into consideration, this figure will be far higher. It is likely therefore that a sizable portion of young people in Ukraine have experienced the loss of a loved one.

While evidence suggests that most young people eventually come to terms with their grief, approximately one-in-ten young people are thought to experience a complicated or prolonged grief reaction, which is linked to functional impairment and manifests as intense preoccupation and/or longing for the deceased, anger, avoidance, sadness, and difficulty accepting the death (Melhem et al., 2011). The potential for such maladaptive grieving responses has been formally recognized by the inclusion of Prolonged Grief Disorder (PGD) in the 11^th^ version of the International Classification of Diseases (ICD-11; World Health Organisation (WHO), 2019). ICD-11 PGD includes ‘core’ and ‘associated’ symptoms, the former reflecting longing for the deceased and persistent preoccupation with the deceased, and the latter reflecting intense emotional pain such as sadness, anger, guilt, and difficulty accepting the loss. These symptoms must be associated with significant functional impairment, be present for at least six months, and exceed societal and cultural standards of grieving to meet diagnostic requirements (WHO, 2019). A recent international review found that the average PGD prevalence rate was 13% among adult populations (Comtesse et al., 2024). Notably, there has been no study which has examined rates of probable ICD-11 PGD in children and adolescents.

Several measures of ICD-11 PGD symptoms exist including the International Prolonged Grief Disorder Scale (IPGDS; Killikelly et al., 2020), the Traumatic Grief Inventory-Self Report Plus (TGI-SR+; Lenferink et al., 2022), the Aarhus Prolonged Grief Disorder Scale (A-PGDs; O’Connor et al., 2023), and most recently is the International Grief Questionnaire (IGQ: Hyland et al., 2023). The IGQ is the focus of the current study and is a short measure of the ‘core’ and ‘associated’ symptoms along with all other diagnostic requirements as outlined in ICD-11. The IGQ was developed as a short measure of ICD-11 PGD which has an equal ratio of core and associated symptoms, ensuring the predictive validity of the defining symptoms (i.e., core symptoms) of the disorder (Hyland et al., 2023). Moreover, the IGQ assesses symptom intensity as opposed to frequency, ensuring that the scale aligns with other measures of stress-related psychopathology and adopts a scoring approach similar to measures of stress-related disorders such as posttraumatic stress disorder (Hyland et al., 2023). The psychometric properties of the IGQ were established in two large samples of bereaved adults from the United Kingdom and Ireland (Hyland et al., 2023). Moreover, the Ukrainian IGQ was recently used to examine the prevalence and correlates of ICD-11 PGD among adults living in Ukraine during the war (Redican et al., 2024). This study found that 11.4% of Ukrainian adults met the diagnostic requirements for probable ICD-11 PGD, and that significant risk factors included recency of loss, death of partner or spouse, loved one dying during the war, lack of contact with deceased prior to their death, and both depression and anxiety. These findings are largely consistent with those observed in the UK and Irish samples (except for death during the war), indicating that the prevalence and correlates of PGD may be consistent across different cultures, countries, and languages.

Given how common bereavement is among young people, it is essential that measures are developed to assess PGD symptoms in children and adolescents. The Traumatic Grief Inventory – Kids – Clinician Administered (Van Dijk et al., 2023) was developed as a clinician administered measure to assess PGD as defined by the DSM-5-TR and ICD-11 in young people. However, there is no self-report measure available which assesses PGD according to the ICD-11 diagnostic requirements. One commonly employed method of assessing psychological distress in young people is to ask parents/caregivers to report of their children’s problems. Although this type of approach has its limitations whereby prior research has shown how parents can underreport stress-related symptoms in their children (Stover et al., 2010). These types of caregiver-report measures can be especially useful in situations where it is not possible or practical to obtain information directly from the young person (e.g., in research it may not be possible to contact children directly, or in practice, clinicians may initially speak to parents and obtain information before a formal assessment with the child). To our knowledge, there is currently no caregiver-report measure of ICD-11 PGD symptoms available to researchers and clinicians.

The goal of this study was to develop a caregiver-report version of ICD-11 PGD symptoms, and to test the psychometric properties of the scores derived from this scale. We adapted the IGQ for this purpose (the new scale is called the IGQ-Caregiver Version, or IGQ-CG), initially developing the scale in English and subsequently translating it into Ukrainian so that we could collect data from a sample of adult parents living in Ukraine (more information on scale development is provided in the Methods section). The situation of an ongoing war in Ukraine provides a uniquely appropriate context in which to screen for bereavement and grief reactions in children via parental reports. In addition to assessing the factorial and concurrent validity, and the internal reliability, of the IGQ-CG scores, we also identified unique correlates of the ICD-11 PGD symptoms and calculated what proportion of young people in Ukraine met diagnostic requirements for probable ICD-11 PGD based on the parental-provided information.

## Methods

### Participants and procedures

Data for the present study was derived from “*The Mental Health of Parents and Children in Ukraine Study: 2023 Follow-up”* study. This project was initially launched in 2022 shortly after Russia’s full-scale invasion of Ukraine to document the social and mental health effects of Russia’s war on Ukraine (Karatzias et al., 2023). Data from an entirely new sample (*N* = 2,050) of adults living in Ukraine was collected online between September 7^th^ and September 18^th^, 2023, by the survey company TGM Research. Participants were drawn from an already-existing panel of research participants that was, prior to the conflict, nationally representative based on the most recent Ukrainian census data. Participants were selected using non-probability-based quota sampling methods to construct a sample that was representative of the adult population of Ukraine with respect to sex, age, and regional. Supplemental Table 1 contains demographics for the full sample including quota variables. Inclusion criteria were that participants be aged 18 years or older, currently living in Ukraine, and able to complete the survey in Ukrainian. Of the total sample, 31.2% (*n* = 639) reported being the parent of a child aged 7–17 years, and these participants were asked to provide information on the mental health of one randomly selected child in their household. This study is based on data collected from these participants.

We limited assessments to children aged 7–17 years based on the availability of measures of psychological distress validated for children between these ages. Parents with more than one child aged 7–17 years were asked to select the child whose birthday was next and complete the questionnaires with that child in mind. The average age of children for whom parents provided information was 11.67 years (SD = 3.13) and the ratio of males (50.5%; *n* = 323) to females (49.5%; *n* = 316) was relatively equal. Most parents (87.6%; *n* = 560) reported that their child lived with them, while a small proportion reported that their child was living with another parent outside of Ukraine (6.6%; *n* = 42) or elsewhere in Ukraine (5.8%; *n* = 37).

### Measures

#### ICD-11 PGD

The IGQ-CG was developed by adapting the wording of the IGQ items to be appropriate for parental/caregiver reports. The wording of the IGQ items was already simple and unambiguous therefore no major changes were made to the IGQ-CG (see Appendix A for the scale). These items were then translated into Ukrainian and back-translated into English to ensure consistency with the original meaning of the items. The translation process was overseen by one of the study authors that is fluent in Ukrainian and English (DM).

Parents were first asked to indicate if their child had ever experienced the death of loved one, and then provided with a list of different people they may have lost (i.e., parent, sibling, grandparent, uncle or aunt, cousin, close friend, other). If selecting multiple people, parents were asked to indicate the loss that they believe affected their child the most and were instructed to answer all questions based on this loss. Parents then indicated how long ago this person died, and the frequency of contact the child had with the deceased in the year prior to death (every day, almost every day, several times a week, several times a month, a few times in the year, not at all during the year).

Parents were then asked to indicate how bothered their child had been by the two ‘core’ (items 1 and 2) and three ‘associated’ (items 3–5) ICD-11 PGD symptoms in the past week using a five-point Likert scale ranging from 0 (‘Not at all’) to 4 (‘Extremely’). Using the response options of ‘No’, ‘Yes’, and ‘I don’t know’, parents were asked whether their child’s symptoms have persisted for longer than would be expected for most people in their social, cultural, or religious setting. Finally, parents were asked to indicate whether these symptoms were causing problems in their child’s life using a ‘Yes’ or ‘No’ response option.

The IGQ-CG can be used to generate symptom severity scores and to identify participants meeting diagnostic requirements for probable ICD-11 PGD. Symptom scores can range from 0 to 20 with higher scores indicating greater symptom severity. To meet diagnostic requirements, a child must be bereaved, the loss must have occurred six months ago or longer, at least one of the two core symptom must be present (endorsement is Likert scale score ≥2), at least one of the three associated symptoms must be present (endorsement is Likert scale score ≥2), the participant must have responded ‘Yes’ or ‘I don’t know’ to the question relating to exceeding expected cultural, social, or religious norms, and functional impairment must be present.

### Predictor variables

#### Demographic variables

Demographic variables included child age (in years) and child sex (1 = male, 2 = female).

#### Loss-related variables

Loss-related variables included time since bereavement (1 = within the last 6 months, 2 = 6 months to a year ago, 3 = 1–2 years ago, 4 = 2–3 years ago, 5 = 3–5 years ago, 6 = 6–10 years ago, 7 = more than 10 years ago), death most affected by (1 = parent, 2 = brother or sister, 3 = grandparent, 4 = uncle or aunt, 5 = cousin, 6 = close friend, 7 = other), and frequency of contact with the deceased in the year prior to their death (1 = every day, 2 = almost every day, 3 = several times a week, 4 = several times a month, 5 = a few times in the year, 6 = not at all during that year). Death most affected by was collapsed into five categories due to low counts (1 = parent, 2 = grandparent, 4 = uncle or aunt, 4 = close friend, 5 = other (other, brother or sister, cousin)).

Parental mental health variables: Parental posttraumatic stress disorder (PTSD) was assessed via the International Trauma Questionnaire (Cloitre et al., 2018). Six items measure PTSD symptoms of re-experiencing, avoidance, and sense of current threat. Parents were asked to indicate how bothered they have been by each of these symptoms in the past month using a five-point Likert ranging from 0 (‘Not at all’) to 4 (‘Extremely’). The ITQ can be used to generate symptom severity scores and to identify participants meeting diagnostic requirements for probable ICD-11 PTSD. Possible scores range from 0 to 30, with higher scores indicating greater symptom severity. The internal reliability of the ITQ scores in the current study was excellent (α = .84). Parental PGD was assessed via the International Grief Questionnaire (IGQ; Hyland et al., 2023). Parents were asked to indicate how bothered they have been by each of these symptoms in the past month using a five-point Likert ranging from 0 (‘Not at all’) to 4 (‘Extremely’). The IGQ can be used to generate symptom severity scores and to identify participants meeting diagnostic requirements for probable ICD-11 PGD. Possible scores range from 0 to 20, with higher scores indicating greater symptom severity. The internal reliability of the IGQ scores in the current study was excellent (α = .87).

### Mental health-variables

#### Generalised anxiety disorder

The Caregiver-Report version of the International Anxiety Questionnaire (IAQ-CG; under review) was completed by all parents. The IAQ-CG is based on the standard IAQ (Shevlin et al., 2023) but the symptom and impairment indicators are modified to be relevant for children (see Appendix A for the items). Parents were asked to indicate how often their child had the symptoms over the last two weeks using a four-point Likert scale ranging from 0 (‘Never’) to 3 (‘Almost always’). Parents were also asked to indicate using ‘yes’ or ‘no’ responses whether these symptoms caused problems in getting along with others, hobbies/fun, school or work, and family relationships.

The IAQ-CG can be used to generate symptom severity scores and to identify participants meeting diagnostic requirements for probable ICD-11 GAD. Symptom scores can range from 0 to 24, with higher scores indicating greater symptom severity. The internal reliability of the IAQ-CG scores in the current study was excellent (α = .88).

#### Depression

The Caregiver-Report version of the International Depression Questionnaire (IDQ-CG; under review) was completed by all parents. The IDQ-CG is based on the standard IDQ (Shevlin et al., 2023) but the symptom and impairment indicators are modified to be relevant for children (see Appendix A for the items). Parents were asked to indicate how often their child has had the symptoms over the last two weeks using a four-point Likert scale ranging from 0 (‘Never’) to 3 (‘Almost always’). Parents were also asked to indicate using ‘yes’ or ‘no’ responses whether these symptoms caused problems with getting along with others, hobbies/fun, school or work, and family relationships.

The IDQ-CG can be used to generate symptom severity scores and to identify participants meeting diagnostic requirements for probable ICD-11 DD. Possible scores range from 0 to 24, with higher scores indicating greater symptom severity. The internal reliability of the IAQ-CG scores in the current study was excellent (α = .89).

#### Psychosocial functioning

The Paediatric Symptom Checklist (PSC-17; Gardner et al., 1999) was completed by all parents. The PSC-17 is a brief measure designed to evaluate a child’s emotional and behavioral problems in the areas of attention, internalizing distress, and externalizing distress. The response structure of the PSC-17 was amended slightly for the present study to capture potential changes in emotional or behavioral problems since the onset of the Russian war, with caregivers rating the presence of symptoms using a three-point Likert scale (0 = Less Often, 1 = The Same, 2 = More Often). The PSC-17 can be used to derive a total scale score (range = 0–34) as well as total scores on the attention subscale (range = 0–10), internalizing subscale (range = 0–10), and externalizing subscale (range = 0–14). The internal reliability of the internalizing subscale (α = .79), externalizing subscale (α = .71), and attention subscale (α = .74) were acceptable.

### Analytic plan

First, the proportion of children that had experienced a lifetime bereavement and the characteristics of these bereavements were reported. Additionally, descriptive statistics for the IGQ-CG item responses were reported.

Second, the latent structure of the IGQ-CG was assessed using confirmatory factor analysis (CFA). The latent structure of the original self-report IGQ (Hyland et al., 2023) was found to be a one-factor model (all items loading onto a single PGD factor) and a correlated two-factor model (items 1 and 2 loading on a ‘core symptoms’ latent factor and items 3-5 loading on an ‘associated symptoms’ latent factor). Thus, we followed the same procedure for the IGQ-CG by testing the fit of one- and two-factor models using robust maximum likelihood (MLR) estimation. Model fit was assessed according to standard recommendations (Hu & Bentler, 1999) where ‘acceptable’ model fit was indicated by a non-significant chi-square value; Comparative Fit Index (CFI; Bentler, 1990) and Tucker-Lewis Index (TLI; Tucker & Lewis, 1973) values ≥.90; and Root Mean Square Error of Approximation (RMSEA; Browne & Cudeck, 1992) and Standardized Root Mean Square Residual (SRMR; Sörbom & Jöreskog, 1981) values ≤.08. These models were tested using Mplus Version 8.9 (Muthén & Muthén, 2017).

Third, following identification of the best fitting CFA model, composite reliability (CR) estimates for the IGQ-CG scores were calculated. Composite reliability estimates are derived from the factor analytic results and provide a more accurate estimation of internal reliability than the more traditionally used Cronbach’s alpha coefficient which assumes that all items load on the same underlying factor equally and without measurement error (Raykov, 1997).

Fourth, correlations between the mental health correlates and the latent variables from the best-fitting factor model were examined. Cohen (2013) conventions were used to interpret effect sizes (.10 = small effect, .30 = moderate effect, .50 = large effect). Moreover, regression analyses were conducted within a structural equation modelling (SEM) framework to examine the relations between the demographic and loss-related variables and the ‘core’ and ‘associated’ latent variables within a regression framework. Finally, the proportion of the sample that satisfied diagnostic requirements for probable ICD-11 PGD was determined as well as in those participants who had experienced a bereavement (i.e., the conditional rate of ICD-11 PGD).

## Results

### Descriptive statistics

Overall, 43.3% (*n* = 277) of parents reported that their child had been bereaved. Table 1 provides the loss-related characteristics of the sample. The most common type of bereavement was the death of a grandparent and most parents reported this as the bereavement which most affected their child. There was significant variation in time since loss, with the modal response being between 3 and 5 years ago. Most parents reported their child was in frequent contact with the deceased prior to their death.

**Table 1.** Loss-related characteristics of the sample (*n =* 277).

	n	%
Type of bereavement		
Parent	21	7.6
Sibling	7	2.5
Grandparent	252	91.0
Uncle or aunt	47	17.0
Cousin	5	1.8
Close friend	17	6.1
Other	32	11.6
Bereavement most affected by		
Parent	19	6.5
Sibling	2	0.7
Grandparent	222	80.1
Uncle or aunt	14	5.1
Cousin	2	0.7
Close friend	10	3.6
Other	9	3.2
Contact with deceased in the year before their death		
Every day	52	18.8
Almost every day	48	17.3
Several times a week	48	17.3
Several times a month	66	23.8
A few times in the year	46	16.6
Not at all during that year	17	6.1
Time since bereavement		
Within the last 6 months	18	6.5
6 months to a year ago	31	11.2
1 – 2 years ago	63	22.7
2 – 3 years ago	37	13.4
3 – 5 years ago	64	23.1
6 – 10 years ago	49	17.7
More than 10 years ago	15	5.4

The IGQ-CG item means and endorsements are presented in Table 2. The observed range of total scores was 0 to 18, with a mean score of 4.20 (SD = 4.02). Endorsement rates ranged from 8.3% (*“They feel guilty or angry about the loss”)* to 30.7% (*“They yearn for the deceased almost every day”)*. A small proportion (3.6%%; *n* = 10), reported that their child’s grief response extended beyond what was considered the norm in their culture, while 16.6% (*n* = 36) reported that they did not know if their child’s grief response extended beyond what was considered the norm in their culture. Moreover, 10.5% (*n* = 29) reported that their child experienced functional impairment due to their grief symptoms.

**Table 2.** Means and endorsement rates for IGQ-CG items (*N =* 277).

Item	Item score		Item endorsement count	
	*M*	*SD*	*n*	%
1. They yearn for the deceased *almost every day*	1.03	0.98	85	30.7
2. They think too much about the deceased *almost every day*	0.89	0.96	73	26.4
3. They feel guilty or angry about their loss	0.32	0.72	23	8.3
4. They have trouble accepting the death of their loved one	1.04	1.10	81	29.2
5. They feel sad or emotionally numb	0.92	1.00	72	26.0
Total	4.20	4.02		
Functional impairment			29	10.5
Cultural criterion			56	20.2

### CFA results and reliability

The CFA results are presented in Table 3. The one-factor model was a poor fit to the data across all fit indices except for the SRMR, whereas the two-factor model was a good fit to the data, with the exception of the RMSEA which was higher than the conventional cut-off for ‘acceptable fit’ of .08. This is likely due to the model having few indicators with high factor loadings (Shi et al., 2020). Thus, it appears that the latent structure of the IGQ-CG is best represented by a correlated two-factor model where the two factors represent the ‘core’ and ‘associated’ symptoms of ICD-11 PGD^1^.

**Table 3.** Fit Statistics for CFA Models (*n* = 277).

Model	x^2^ (df), *p*	TLI	CFI	RMSEA (90%C.I.)	SRMR
One-factor model	84.823 (5), *p* < .001	.716	.858	.240 (.197, .286)	.051
Two-factor model	15.071 (4), *p* = .005	.951	.980	.100 (.050, .156)	.017

*Note.* x^2^ = chi-square test, TLI = Tucker Lewis Index, CFI= Comparative Fit Index, RMSEA = Root Mean Square Error of Approximation, Standardized Root Mean Square Residual.

The core and associated symptom latent variables were positively and strongly correlated (r = .85, *p* < .001), and all items, with the exception of item 3 ‘They feel guilty or angry about their loss’, loaded on their respective factor strongly, positively, and significantly. The internal reliability of the core (CR = .88) and associated (CR = .60) items scale scores were adequate, as was the reliability of all scale items (CR = .89). Factor loadings are provided in Supplemental Table 2.

### Correlation and regression results

Table 4 provides correlations between each of the predictor variables and the ‘core symptoms’ and ‘accessory symptoms’ latent variables. There were significant, small to moderate associations between internalizing problems, depression symptoms, anxiety symptoms and both the core symptoms and accessory symptoms latent variables.

**Table 4.** Correlations between the ‘core symptoms’ and ‘accessory symptoms’ latent variables and mental health correlates (*n =* 277).

Latent variable	Anxiety (IAQ-CG)	Depression (IDQ-CG)	Internalizing problems (PSC-17)	Externalizing problems (PSC-17)	Attentional problems (PSC-17)
Core symptoms	.28***	.22***	.24***	.09	.21
Accessory symptoms	.36***	.35***	.36***	.13*	.14

*Note.* *p < .05, **p < .01, ***p < .001.

The standardized regression coefficient between each correlate and the ‘core symptoms’ and ‘accessory symptoms’ latent variables are reported in Table 5. In the bivariate analyses, significant negative predictors of the core symptoms latent variable and accessory symptoms latent variable included time since bereavement and frequency of contact with the deceased in the year prior to their death, while significant positive predictors of both latent variables included higher levels of parental PTSD symptoms and parental PGD symptoms. A significant positive predictor of the accessory symptoms latent variable was the death of a close friend (as compared to grandparent). These associations remained the same in the multivariate analyses with the exception of parental PGD symptoms which became non-significant.

**Table 5.** Standardized Regression Coefficients From Bivariate and Multiple Regression Analysis of ‘Core Symptoms’ and ‘Accessory Symptoms’ Latent Variables (*n* = 277).

	*Bivariate*		*Multivariate*	
	*Core*	*Accessory*	*Core*	*Accessory*
Age	.04	.08	.06	.08
Sex (females)	−.01	.01	−.01	.02
Time since bereavement	−.21**	−.19**	−.16**	−.14*
Contact with deceased during year prior to death	−.27***	−.23***	−.17**	−.13*
Death most affected by				
Parent	.08	.11	.02	.06
Grandparent	-		-	-
Uncle or aunt	.06	.06	.04	.04
Close friend	.04	**.18**	.01	.16*
Other	−.04	.06	−.04	.05
Parental PGD	.48***	.46***	−.03	.04
Parental PTSD	.21**	.26***	.44***	.40***
R-squared			**.24**	**.25**

*Note.* *p < .05, ***p < .01, ***p < .001.

### Parental-reported prevalence estimates of probable ICD-11 PGD

Overall, 1.4% (95% CI = 0.5%, 2.3%) of children met the diagnostic requirements for probable ICD-11 PGD based on parental-reported information, and amongst those with a lifetime bereavement, the conditional rate was 3.2% (95% CI = 1.2%, 5.4%).

## Discussion

The objectives of this study were to develop the IGQ-CG, a caregiver-reported version of the IGQ, and evaluate the psychometric properties of the Ukrainian version of the IGQ in Ukrainian children and adolescents. Findings from the current study provide support for the validity and reliability of the IGQ-CG, while demonstrating how the IGQ-CG identifies a small number of young people with clinically significant symptoms of prolonged grief.

Aligning with research on the adult IGQ (Hyland et al., 2023), a correlated two factor model of the IGQ-CG was found to be an excellent representation of the latent structure of the scale, while the internal reliability of the accessory items was low. This will be monitored in future studies. Notably, correlations between the core symptoms and accessory symptoms latent variables were high (>.85) in the two-factor model, indicating poor discrimination between the latent variables. While the latent variables demonstrated criterion validity through associations with measures of depression, anxiety, and internalizing problems, the magnitude of associations between the mental health variables and the core symptoms and accessory symptoms latent variables were similar, indicating that no substantial unique predictive utility was being obtained from using subscale scores. Therefore, these results suggest that overall scores may be useful for analytical reasons; if a more detailed examination is needed, the subscale scores may prove beneficial. However, overall scores should be used cautiously given the one-factor model’s poor fit, and it may be more appropriate to use separate core-accessory severity scores.

Findings demonstrated how parental PTSD symptoms and PGD symptoms were robust predictors of PGD symptoms in young people. This is not necessarily surprising given that parental PTSD has been linked to psychological distress in their offspring through mechanisms such as disrupted family functioning, genetics, and other challenges associated with living with a parent with mental health problems (Lambert et al., 2014), while PGD has been shown to transmit from parents to their adult children (Lenferink & O’Connor, 2023). However, prior research has shown how parental psychopathology can influence their reporting of their child’s psychopathology (Maoz et al., 2014). Thus, it may be that in the context of parental PTSD or PGD, caregiver reports of child psychopathology may be influenced by attentional bias towards threat and negative emotions, leading to potential overreporting of child psychopathology (Creech & Misca., 2017; Dekel & Monson, 2010). Notably, the fact that parental psychopathology is a key predictor of child PGD scores does not undermine the validity of the IGQ-CG. Specifically, during the development of the IGQ-CG this caveat was acknowledged but it was deemed that the potential benefits from developing this measure (e.g., being useful in situations where it is not possible or practical to obtain information directly from the young person) outweighed this drawback.

Consistent with research indicating that psychological issues in bereaved young people are evident in the first two years post-bereavement (Pham et al., 2018), the current study showed how PGD symptoms were highest in those who experienced a more recent bereavement. This is an important finding since there are not many studies examining young people’s grief responses over time, which makes it challenging to establish the optimal length of time after bereavement to be able to diagnose a young person with PGD (Kaplow et al., 2012). Furthermore, consistent with research demonstrating closeness to the deceased as an established risk factor of PGD (e.g., Holland & Neimeyer, 2011; Sekowski & Prigerson, 2022), our findings showed how being in more frequent contact with the deceased prior to their death was associated with the highest levels of PGD symptoms. Relationship to the deceased was generally unrelated to the core symptoms and accessory symptoms latent variables, but results showed that the death of a close friend was linked to higher scores on the accessory symptoms latent variable (in contrast to the death of a grandparent). It is likely that the death of a friend during childhood can be considered as one of the most unexpected and difficult to understand types of loss. Indeed, common reactions to the death of a friend in childhood include disbelief, fear, anger, self-blame, guilt, loneliness, concentration difficulties, among others (Cowan, 2010).

Finally, findings demonstrated that 3.2% of bereaved young people met diagnostic requirements for probable PGD. Given that there are currently no studies providing prevalence rates of probable PGD (according to either ICD-11 or DSM-5-TR requirements), it is difficult to ascertain whether these rates are considered high in the context of childhood bereavement. Thus, it is crucial to establish an evidence base pertaining to the prevalence of probable PGD in children and adolescents particularly given that comparisons to adult populations may not be accurate. Specifically, among young individuals, the symptom of preoccupation—that is, thinking excessively about the deceased—often takes the form of distress when the young person is separated from their loved one’s possessions (Kentor & Kaplow, 2020). Thus, while an adult’s manifestation of this symptom may be more cognitive, a young person’s manifestation of this symptom may be more behavioural. Because of these types of variations in symptom presentation between youth and adults, Kaplow et al. (2012) proposed several developmental factors that should be taken into consideration when diagnosing any youth-specific grief-related disorder. These included: (1) considering a child’s grieving process within the context of their caregiving environment; (2) adding a “developmental variation” criterion to the cultural criterion, requiring the bereavement response to defy age-appropriate norms; (3) amending symptom descriptions to account for developmental stage; and (4) including as a symptom the excessive concern that bereaved young people frequently have for the well-being of their surviving caretaker (Kaplow et al., 2012). Consequently, considering that PGD was just recently included to the diagnostic nomenclature, it is imperative to ascertain the validity of the disorder in young people and whether the ICD-11 needs to include a developmentally sensitive version of the disorder.

There are several limitations associated with the current study that should be taken into consideration. First, given the ongoing conflict and the mass displacement of people in Ukraine, it was not possible to obtain a representative sample. The sample size of young people in the current study is relatively small and thus, findings may not generalize to the entire youth population of Ukraine. Second, the present study is based on parental reports. There is research illustrating discrepancies in reporting of mental health problems between parents and their children, with parent-child relationship and family structure representing important predictors of disagreement (Van Roy et al., 2010). This is especially pertinent to the PGD context, since the ICD-11 emphasises that children will frequently exhibit behavioural symptoms rather than verbally describing feelings of longing for the deceased or a persistent preoccupation with thoughts of the deceased (WHO, 2019). Given that the majority of the IGQ questions are cognitive and emotional in nature, parents might not be able to adequately answer them on behalf of their children. This also highlights the need for further investigation of IGQ-CA self-reported measures both within Ukraine and globally, as well as the discrepancies between parental and child responses as well as across different countries and cultural contexts. Moreover, because relatively few young individuals fulfilled the diagnostic criteria for probable PGD, the current study was unable to investigate factors associated with meeting these requirements. However, other studies have demonstrated that, for young individuals, a dimensional approach to maladaptive grieving responses outperforms the categorical approach (Melhem et al., 2013). Finally, similar to the IGQ, some items in the IGQ-CG make reference to different kinds of emotional distress thereby prohibiting the identification of specific aspects of the grief response and would not capture the breadth of the grief response (Hyland et al., 2023). For a more precise evaluation of symptoms and a broader assessment, a clinical interview would prove necessary. Notably, no clinical interview is yet available.

In summary, preliminary findings suggest that the IGQ-CG is a valid and reliable measure of ICD-11 PGD symptoms in children and adolescents. This study highlights that one-in-four young people are affected by bereavement and that 3.2% of bereaved young people meet diagnostic requirements for probable PGD. Identification of correlates associated with greater core and accessory PGD symptoms will contribute toward the formulation of targeted guidelines for the assessment, treatment, and prevention of PGD in young people. This is especially crucial in the context of Ukraine, where the war will regrettably cause a significant number of deaths.

## Supplemental Material

Supplemental Material - Development and validation of the caregiver-report version of the international grief questionnaire (IGQ-CG): Results from a Ukrainian sample of parents Supplemental Material for Development and validation of the caregiver-report version of the international grief questionnaire (IGQ-CG): Results from a Ukrainian sample of parents by Enya Redican, Mark Shevlin, Philip Hyland, Thanos Karatzias, Dmytro Martsenkovskyi, and Menachem Ben-Ezra in Clinical Child Psychology and Psychiatry

## Note

1. These CFA models were also tested using the “binary scoring” method as the IGQ-CV can be used to screen for probable ICD-11 PGD. The two-factor model was also found to provide the best fit to the data The incremental fit indices (TLI, CFI) provided a ‘perfect’ fit, however, this should be interpreted with caution given the small sample size and the small proportion of participants who met diagnostic requirements. Factor loadings using the binary scores ranged from .85 for item 3 to .98 for item 1 and item 2. There was also a strong correlation between the ‘core symptoms’ and ‘accessory symptoms’ latent variables using the binary items scores, r = .91. See Supplemental Table 3 for model fit statistics.
