# Supplementary material for: Development and validation of the caregiver-report version of the international grief questionnaire (IGQ-CG): Results from a Ukrainian sample of parents
Source: Clin Child Psychol Psychiatry. 2024 Jun 13;29(4):1481–96. doi: 10.1177/13591045241260897 (PMC11475617; doi:10.1177/13591045241260897)
Supplement: Supplemental Material - Development and validation of the caregiver-report version of the international grief questionnaire (IGQ-CG): Results from a Ukrainian sample of parents [file sj-pdf-1-ccp-10.1177_13591045241260897.pdf]

Supplementary 1

The International Grief Questionnaire – Caregiver Version (IGQ-CG)

Has anyone close to the child you care for ever died (e.g., a parent, grandparent, sibling, close friend)?

Yes (1) No (2)

How long ago did this person die?

a) Within the last 6 months (1)
b) 6 months to a year ago (2)
c) 1-2 years ago (3)
d) 2-3 years ago (4)
e) 3-5 years ago (5)
f) 6-10 years ago (6)
g) More than 10 years ago (7)

Below are a number of problems that people sometimes report following the death of a person close to them. Using the scale below, please choose the answer that best describes how **bothered the child you care for has been by each of these problems in the past week.**

Not at all (0)	A little bit (1)	Moderately (2)	Quite a bit (3)	Extremely (4)

PGD_1: They yearn for the deceased almost every day.	0	1	2	3	4
PGD_2: They think too much about the deceased almost every day.	0	1	2	3	4
PGD_3: They feel guilty or angry about their loss.	0	1	2	3	4
PGD_4: They have trouble accepting the death of their loved one.	0	1	2	3	4
PGD_5: They feel sad or emotionally numb.	0	1	2	3	4

Would you say these problems have been going on for longer than most people in your social, cultural, or religious context would expect?

- No (0)
- Yes (1)

Have these experiences caused problems in the child's life?

- No (0)
- Yes (1)

SPSS scoring: Probable Diagnosis

```
IF (PGD_1 GE 2) or (PGD_2 GE 2) IGQ_Core_dx=1.
IF (PGD_3 GE 2) or (PGD_4 GE 2)  or (PGD_5 GE 2)  IGQ_Acc_dx=1.
EXECUTE.
IF  (Loss = 1) and  (LongAgo GE 2) and (IGQ_Core_dx=1) and (IGQ_Acc_dx=1)  and (FI = 1) and (Culture GE 1) NEW_IGQ_dx=1.
Execute.

RECODE NEW_IGQ_dx (MISSING=0).
EXECUTE.
```

SPSS scoring: Dimensional Scoring

```
Compute Core_tot = PGD_1 + PGD_2.
Compute Acc_tot = PGD_3 + PGD_4 + PGD_5.
Compute PGD_tot = PGD_1 + PGD_2 + PGD_3 + PGD_4 + PGD_5.

EXECUTE.
```

Supplementary Table 1.

Comparison between census data and sample composition across the three quota variables for sample selection.

	Census figures	Sample composition
Sex		
Male	50.0%	51.7%
Female	50.0%	48.3%
Age		

18-29	16.0%	20.8%
30-39	21.0%	25.2%
40-49	19.0%	23.2%
50-59	17.0%	19.0%
60 and older	26.0%	11.8%
Region		
Western Ukraine	26.0%	24.3%
North Ukraine	19.0%	22.0%
Central Ukraine	14.0%	13.5%
Eastern Ukraine	18.0%	15.6%
South Ukraine	23.0%	24.7%

Supplementary Table 2: Standardized factor loadings and standard errors for the two-factor model

		Core	Ac
1.	They yearn for the deceased almost every day.	.955	
2.	They think too much about the deceased almost every day.	.915	
3.	They feel guilty or angry about their loss.		
4.	They have trouble accepting the death of their loved one.		
5.	They feel sad or emotionally numb.		

Note: All loadings significant at p < .001.

Model	χ^2 (df), *p*	TLI	CFI	RMSEA (90% C.I.)	SRMR
One-factor model	17.533 (5) , p = .004	.995	.997	.095 (.049, .145)	.035
Two-factor model	1.33 (4), p = .857	1.00	1.00	.000 (.000, .049)	.008

Supplementary Table 3: Fit Statistics for CFA models using ‘binary scoring’ method

Note: χ^2 = chi-square test, TLI= Tucker Lewis Index, CFI= Comparative Fit Index, RMSEA= Root Mean Square Error of Approximation, Standardized Root Mean Square Residual.
